# An Adaptive Compression Method for Lightweight AI Models of Edge Nodes in Customized Production

**DOI:** 10.3390/s26020383

**Published:** 2026-01-07

**Authors:** Chun Jiang, Mingxin Hou, Hongxuan Wang

**Affiliations:** 1 School of Mechanical Engineering, Guangdong Ocean University, Zhanjiang 524088, China; chjiang@gdou.edu.cn; 2 Guangdong Key Laboratory of Precision Equipment and Manufacturing Technology, South China University of Technology, Guangzhou 510641, China; 3 Guangdong Engineering Technology Research Center of Small Household Appliances Innovation Design and Manufacturing, Zhanjiang 524088, China; 4 South Subtropical Crops Research Institute, Chinese Academy of Tropical Agricultural Sciences, Zhanjiang 524003, China; wanghongxuan@catas.cn

**Keywords:** edge intelligence, model compression, adaptive optimization, RL, customized production line

## Abstract

In customized production environments featuring multi-task parallelism, the efficient adaptability of edge intelligent models is essential for ensuring the stable operation of production lines. However, rapidly generating deployable lightweight models under conditions of frequent task changes and constrained hardware resources remains a major challenge for current edge intelligence applications. This paper proposes an adaptive lightweight artificial intelligence (AI) model compression method for edge nodes in customized production lines to overcome the limited transferability and insufficient flexibility of traditional static compression approaches. First, a task requirement analysis model is constructed based on accuracy, latency, and power-consumption demands associated with different production tasks. Then, the hardware information of edge nodes is structurally characterized. Subsequently, a compression-strategy candidate pool is established, and an adaptive decision engine integrating ensemble reinforcement learning (RL) and Bayesian optimization (BO) is introduced. Finally, through an iterative optimization mechanism, compression ratios are dynamically adjusted using real-time feedback of inference latency, memory usage, and recognition accuracy, thereby continuously enhancing model performance in edge environments. Experimental results demonstrate that, in typical object-recognition tasks, the lightweight models generated by the proposed method significantly improve inference efficiency while maintaining high accuracy, outperforming conventional fixed compression strategies and validating the effectiveness of the proposed approach in adaptive capability and edge-deployment performance.

## 1. Introduction

The rapid evolution of intelligent manufacturing and industrial automation has generated unprecedented demand for low-latency, high-efficiency, and energy-constrained AI capabilities on edge devices [1,2]. As production scenarios shift from large-scale standardized workflows to flexible, customized, and small-batch manufacturing, intelligent perception and decision making increasingly need to be performed at the network edge to ensure real-time responsiveness and operational stability. Edge intelligence, characterized by on-device inference, reduced reliance on network communication, and real-time control, has therefore become a key enabler of modern smart factories and Industry 4.0 systems [3,4,5,6]. In particular, deep learning-based vision and sensing models deployed on distributed edge nodes play a vital role in applications such as defect detection, quality inspection, robotic manipulation, and adaptive process control. However, deploying these models in customized production lines remains challenging due to limited computational resources, memory constraints, heterogeneous hardware platforms, and stringent real-time requirements.

Traditional model compression techniques, including pruning, quantization, low-rank decomposition, and knowledge distillation, have been extensively investigated to reduce model size and computational overhead [7]. However, most existing approaches rely on fixed or manually tuned compression configurations, which often fail to generalize across different tasks, datasets, and hardware platforms. In customized manufacturing environments, task diversity is substantially higher than that in conventional mass production workflows. For instance, production lines may switch among defect detection, object localization, pattern recognition, and tool monitoring tasks, each with distinct accuracy requirements and latency constraints [8]. At the same time, edge devices may vary significantly in terms of memory capacity, computational resources, power budgets, and thermal constraints. Under such dynamic and heterogeneous conditions, static compression schemes frequently fail to achieve optimal performance, resulting in unacceptable accuracy degradation, excessive inference latency, or unstable runtime behavior [9,10].

Moreover, conventional lightweighting methods generally lack effective mechanisms to incorporate real-time performance feedback from practical industrial operations. As manufacturing tasks evolve or sensor inputs vary, the optimal compression ratios and strategy combinations, such as pruning rates, quantization bit width, and distillation strengths, may change over time [11,12]. Without adaptive evaluation and adjustment mechanisms, lightweight models are prone to performance drift, which undermines their reliability in continuous industrial deployment. This issue becomes more pronounced when production lines frequently switch tasks or adopt new process specifications, requiring the edge AI system to rapidly reconfigure in order to maintain high accuracy and responsiveness.

Recent academic research has begun exploring dynamic and context-aware model compression. However, most existing approaches typically optimize a single compression strategy, such as RL-based pruning, quantization-aware training, or dynamic bitwidth assignment, and lack comprehensive integration of multiple compression strategies [13]. Furthermore, the majority of adaptive methods are designed for general-purpose mobile or Internet of Things applications rather than for the highly constrained, deterministic, and reliability-critical environment of customized industrial manufacturing. The absence of a unified framework that jointly accounts for task requirements, hardware constraints, multi-strategy compression, and real-time performance feedback significantly limits the practical adoption of adaptive model compression in real-world production systems.

Existing methods are unable to adaptively compress models for diverse tasks and heterogeneous edge devices while maintaining real-time performance. To overcome these limitations, this paper proposes a novel adaptive multi-strategy compression method for edge intelligence in customized manufacturing, integrating RL with BO.

The main contributions of this work are summarized as follows: First, a task aware and hardware aware model compression framework is proposed for customized manufacturing scenarios, which enables the dynamic alignment of compression configurations with production requirements and device capabilities, and incorporates a multi-strategy compression candidate pool that integrates pruning, quantization, and distillation to exploit complementary strengths and enhance robustness across heterogeneous edge environments. Second, a hybrid RL and BO-based decision engine is developed to autonomously generate near-optimal compression strategies, balancing exploration efficiency and deployment effectiveness. Third, a real-time closed-loop feedback mechanism is introduced to continuously monitor inference performance on edge devices and adaptively adjust compression ratios, thereby ensuring long term model stability under dynamic manufacturing conditions.

Section 2 reviews related work on model compression techniques and edge intelligence deployment in industrial environments. Section 3 introduces the proposed adaptive compression framework. Section 4 elaborates on the mathematical formulation and compression strategy. Section 5 presents experimental results and performance analysis. Section 6 concludes the paper.

## 2. Related Work

The proposed adaptive multi-strategy model compression method is grounded in several research domains, including static model compression, dynamic model compression, and edge-intelligence deployment in industrial environments. This section provides a comprehensive review of research domains and discusses their limitations in the context of customized manufacturing tasks.

Static model compression has long been recognized as an effective strategy for reducing the size and computational cost of deep neural networks prior to deployment. Classical techniques include pruning, quantization, low-rank decomposition, and knowledge distillation, each contributing to model lightweighting from different perspectives [14]. Pruning selectively removes redundant neurons, channels, or weights from trained neural networks. Early studies [15,16], such as weight pruning and unstructured sparsification, demonstrated substantial parameter reduction; however, these methods often produce irregular sparsity patterns that are inefficient for edge hardware execution. Subsequent works [17,18] shifted toward structured pruning, in which entire channels or blocks are removed to ensure compatibility with GPUs and embedded accelerators. Despite these advantages, structured pruning typically requires manual tuning of pruning ratios, which limits adaptability and generalization across heterogeneous edge environments. Quantization reduces model size and computational complexity by representing network parameters and activations using lower-precision numerical formats, such as 8-bit integers. Quantization-aware training and post-training quantization have both achieved significant success, enabling efficient inference on microcontrollers and low-power GPUs [19]. Nevertheless, quantization inherently involves a trade-off between precision and efficiency, which must be carefully balanced. Without task-specific adaptation, fixed bitwidth quantization may lead to unpredictable accuracy degradation under diverse manufacturing scenarios. Knowledge distillation (KD) transfers knowledge from a large teacher network to a lightweight student model, thereby improving generalization and robustness, particularly in lightweight architectures. Although KD can complement pruning and quantization, the teacher–student paradigm typically relies on offline training and manual configuration [20]. In dynamic or multi-task industrial settings, this fixed design limits responsiveness to task changes, rendering KD insufficient as a standalone adaptive compression technique. Despite significant progress in static compression methods, several inherent limitations remain. Fixed compression configurations constrain adaptability, and the lack of explicit consideration for hardware constraints often results in suboptimal performance on heterogeneous edge devices. These challenges underscore the need for dynamic and context-aware compression methods in customized manufacturing environments.

Dynamic compression techniques adjust model architecture or numerical precision in response to runtime conditions or input characteristics. These methods [7,21] aim to enhance generalizability under varying deployment requirements. Elastic neural networks enable the selective activation of network components based on input complexity or hardware load. Early studies [22,23,24], including runtime pruning, dynamic channel selection, and conditional computation, demonstrated notable improvements in computational efficiency. However, these methods are primarily input-adaptive rather than task- adaptive, and they often require modifications to network architecture, which limits their applicability to practical industrial models. Dynamic quantization methods adapt bit widths during inference or in response to workload variations. Several studies [25,26] have investigated mixed precision quantization guided by RL or heuristic search. Despite achieving performance gains, these methods typically fail to incorporate broader task-level constraints, such as accuracy thresholds, latency budgets, and energy limits, which are essential in customized manufacturing environments. A limited number of frameworks [27,28] attempt to adjust distillation strength during deployment, but the majority rely on static teacher–student architectures. Runtime KD requires complex coordination between teacher and student models and introduces additional computation overhead, rendering it impractical for real-time industrial applications. Dynamic methods offer important advances but remain constrained in industrial settings. Single strategy optimization, insufficient consideration of hardware heterogeneity, reliance on heuristic rules, and the absence of real-time feedback mechanisms collectively limit adaptability and long term reliability. These limitations motivate the need for a multi-strategy, reinforcement-driven compression method.

Recent advances in edge computing have stimulated substantial research on deploying AI directly on industrial devices. Cloud-based defect detection methods often suffer from excessive latency and limited scalability in industrial environments, motivating recent studies to develop lightweight edge-deployable vision models using quantization and pruning to improve efficiency [29,30]. Despite these advances, existing solutions remain constrained by fixed compression strategies and lack adaptive mechanisms suitable for heterogeneous and dynamically evolving production environments. Xie et al. [31] investigated serverless edge computing for Industrial Internet of Things applications, focusing on workflow scheduling for cloud-edge collaborative processing under resource constraints and employing deep RL-based scheduling to improve service quality. Halenar et al. [32] developed an edge-enabled system for production equipment condition assessment by processing sensor layer data with artificial intelligence and expert systems, enabling on-device analysis and trigger generation for predictive maintenance. Surabhi [33] examined the role of edge computing and advanced sensing in real-time quality control for Industry 4.0, demonstrating that the integration of digital imaging and AI-driven defect detection can enable near-zero defect additive manufacturing. The study further proposed a predictive maintenance framework that combines digital twins, advanced data processing, and AI algorithms to enhance product lifecycle resilience and reduce high-cost failures in modern manufacturing systems. Huang et al. [34] introduced RecSLAM, a multi-robot SLAM system that leverages a robot–edge–cloud architecture and hierarchical map fusion to overcome the computational limits of robots and the communication overhead of cloud offloading. Despite these contributions, no existing work provides a unified, adaptive, multi-strategy compression solution specifically designed for customized manufacturing environments with closed-loop feedback.

The review highlights two key research gaps. First, existing work lacks an integrated and adaptive compression framework that unifies multiple strategies and adapts to diverse manufacturing tasks and heterogeneous edge hardware platforms. Second, most current approaches fail to incorporate effective closed-loop optimization, as they rarely integrate RL and BO or leverage runtime feedback to maintain stable performance during deployment. These gaps motivate the proposed adaptive multi-strategy compression method for lightweight AI models of edge nodes in customized production.

## 3. System Framework

**Figure 1:**
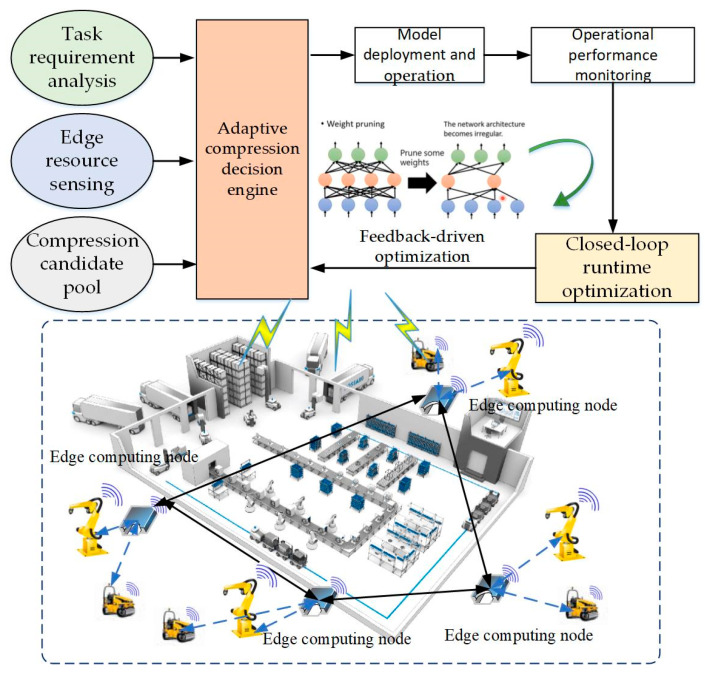
Customized production edge model compression framework.

The proposed adaptive multi-strategy compression framework is designed to address the stringent requirements of customized manufacturing environments, where edge AI systems must operate under strict constraints of latency, accuracy, memory, and energy consumption, as shown in Figure 1. The proposed framework comprises five tightly integrated layers. These layers include a task requirement analysis layer, an edge resource sensing layer, a multi-strategy compression candidate pool layer, an adaptive compression decision engine layer, and a closed-loop runtime optimization layer. Industrial inspection tasks vary considerably across production lines, exhibiting different accuracy thresholds, acceptable latency bounds, and power budgets. The task requirement analysis layer extracts these explicit constraints and encodes them into a formalized task requirement vector. Beyond these explicit constraints, this module further analyzes the statistical characteristics of the manufacturing data, such as object complexity, defect frequency, and class imbalance. Such profiling enables the system to estimate the inherent difficulty of the task, which guides the compression process by preventing over-aggressive reduction that might compromise essential discriminative features. Edge devices deployed in industrial environments often exhibit substantial heterogeneity in hardware capabilities, arising from differences in deployment ages, cost constraints, and performance tiers. The edge resource sensing layer performs continuous hardware introspection to capture CPU and GPU computational capability, available memory, communication bandwidth, and power limitations. The collected information is formalized into a unified hardware resource descriptor. In addition, the edge resource sensing layer monitors runtime variations, such as thermal throttling and dynamic memory pressure, enabling real-time adaptation to actual operating conditions rather than relying solely on static device specifications.

To support flexible adaptation, the framework incorporates a comprehensive pool of compression strategies, including structured pruning for channel-level reduction, low-bit quantization for computational efficiency, low-rank decomposition for dimensionality reduction, and knowledge distillation for recovering model accuracy after aggressive compression. Each strategy is parameterized to form a high-dimensional search space of candidate compression configurations. This diverse strategy pool serves as the foundation for generating tailor-fit lightweight models optimized for both the task requirements and hardware constraints. At the core of the framework lies a hybrid decision engine that integrates BO for global exploration with RL for online refinement. BO identifies promising regions in the strategy space using a Gaussian Process surrogate model, significantly reducing the sampling cost required to locate high-performance configurations. These candidate strategies are used as initialization seeds for the RL agent, which continuously adjusts compression parameters based on real-time feedback. The RL agent interprets accuracy, latency, memory usage, and energy metrics as its state, and outputs fine-grained adjustments to pruning ratios, quantization bit widths, and distillation strengths. This hybrid design enables both rapid convergence and stable long-term adaptation. Furthermore, industrial environments are inherently dynamic, exhibiting frequent changes in lighting conditions, object types, motion patterns, and sensor noise.

The closed-loop runtime optimization layer forms the feedback backbone of the system. It continuously monitors inference performance, compares it against task constraints, and triggers corrective actions through the decision engine. The closed-loop design prevents performance degradation over long operating periods and ensures that the deployed model remains aligned with evolving production conditions. Together, these layers form a unified architecture capable of delivering lightweight, high-performance AI models that self-adjust to task variability and hardware heterogeneity, making the framework particularly suitable for Industry 4.0 edge intelligence applications. It is important to clarify that the proposed AMS-RLBO framework is primarily built upon post hoc model compression techniques, which can be applied to pre-trained models without requiring frequent retraining. Although optional offline fine-tuning or knowledge distillation may be used during the initial deployment stage to recover potential accuracy loss, the runtime closed-loop optimization mechanism does not involve model retraining. Instead, it dynamically adjusts compression configurations based on real-time performance feedback, making the framework suitable for industrial scenarios where retraining is costly.

## 4. System Model and Method

### 4.1. Task Requirement Analysis Model

**Figure 2:**
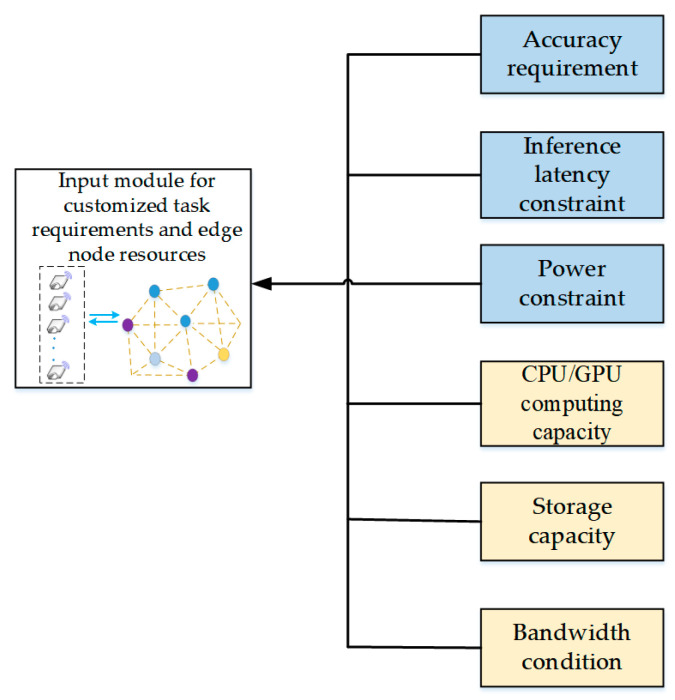
Mapping between customized production tasks and edge resource awareness.

In customized manufacturing scenarios, different processing stages, product categories, and edge nodes exhibit distinct performance requirements and resource constraints. To ensure interpretability, controllability, and computational tractability in the model compression process, a task requirement analysis model (TRAM) was developed. The mapping between customized production tasks and edge resource awareness is shown in Figure 2. This model incorporates multiple constraints, structural dimensions, and feature levels. By using task inputs, performance objectives, resource boundaries, and dynamic environmental factors as its core descriptive variables, TRAM provides a formalized representation of task requirements in industrial production environments.

Task requirements in customized manufacturing environments are derived from three major sources: process specifications such as accuracy thresholds for detection, segmentation, or recognition; system timing constraints, including the maximum permissible inference latency and periodic real-time execution requirements; and energy budgets that define limits on per-task consumption and peak power usage. To enable effective downstream optimization, these raw requirements are standardized through min–max normalization to reconcile heterogeneous units, discrete task categories are encoded as high-dimensional sparse vectors using one-hot representations or semantic embeddings, and temporal requirements such as periodically varying workloads are converted into sliding-window features to capture their dynamic behavior.

To formulate accuracy, latency, and energy consumption as optimizable objectives, a multi-dimensional performance constraint vector $C=\left\{C_{acc},C_{lat},C_{pow}\right\}$ is defined. Here, *C_acc_* represents the minimum acceptable accuracy, *C_lat_* denotes the upper bound on inference latency, and *C_pow_* specifies the maximum allowable energy consumption per inference. A penalty function is further introduced to quantify the degree of constraint violation, expressed as:

(1) $$P\left(C\right)=\sum_{i}\lambda_{i}\cdot \max(0,f_{i}-C_{i}),$$

where *f_i_* denotes the measured performance and *λ_i_* controls the weight of each constraint. This formal representation ensures that subsequent compression-decision algorithms operate within a differentiable or approximately differentiable space, enabling effective multi-objective optimization.

Since a single edge node may execute multiple tasks such as detection, tracking, and quality analysis, TRAM employs a graph-based representation, referred to as the task dependency graph (TDG) [35], to model inter-task relationships. In this structure, nodes correspond to independent inference tasks, edges encode temporal or data dependencies as well as composite operational requirements, and edge weights reflect the degree of data coupling or the extent of shared submodules. This formulation captures parallel, hierarchical, and tightly coupled task patterns, enabling the compression framework to determine whether shared components should be assigned unified or task-specific compression ratios.

To ensure consistency between task requirements and actual hardware capabilities, hardware resources are represented as a resource vector $R=\left\{R_{cpu},R_{gpu},R_{mem},R_{bw}\right\}$, with each component updated in real time. In addition, dynamic operational factors are incorporated, including the current load of the edge node, variations in task execution cycles, hardware temperature and power-limit effects, and network bandwidth fluctuations such as those caused by wireless instability. These elements are integrated into an extended constraint space $\Omega =\left\{C,R,TDG,E\right\}$, where *E* denotes the set of environmental dynamics. This unified representation provides the decision engine with a comprehensive view of both static constraints and runtime variations, enabling adaptive and hardware-aware strategy selection.

The above information is encoded into a compact task embedding vector that could be consumed by the decision engine, formulated as:

(2) T=F(C,R,TDG,E).

In Equation (2), the function *F* denotes a feature composition and embedding mapping process rather than a fixed analytical expression. Specifically, *F* takes as input the normalized performance constraints, encoded task attributes, task dependency graph features, and real-time hardware/resource states, and first concatenates these heterogeneous features into a unified representation. Subsequently, a lightweight nonlinear mapping function is applied to transform the concatenated features into a fixed-dimensional task embedding vector. This design ensures compatibility with downstream RL and BO modules, while maintaining low computational overhead suitable for edge deployment.

The mathematical representations defined in the TRAM are explicitly transformed into numerical constraints that guide the RL process. First, the task embedding vector generated in Equation (2) is directly used as part of the RL state representation, allowing the policy network to perceive task-specific accuracy thresholds, latency limits, energy budgets, and hardware conditions. Second, the performance constraint vector and the associated penalty function in Equation (1) are incorporated into the reward formulation, where constraint violations are penalized to discourage infeasible compression strategies. Finally, hard constraint violations, such as exceeding maximum latency or energy budgets, are treated as termination conditions that end the current episode early. Through this state–reward–termination coupling, the abstract mathematical definitions in TRAM are concretely instantiated as numerical signals that govern the optimization behavior of the RL agent. Through this embedding formulation, heterogeneous task constraints and system states are represented in a unified and compact form, enabling seamless integration with RL and BO modules.

### 4.2. Edge Resource Description Model

In a lightweight model-compression method for customized production lines, a precise and structured representation of edge-node hardware resources is regarded as a prerequisite for enabling the adaptive decision engine to perform compression-strategy search. To ensure portability and reliability of compression strategies across heterogeneous device environments, a structured description process for hardware resources is constructed on top of the fused model of hardware attributes and dynamic factors introduced in the previous section. This process is defined through metric quantification, normalized modeling, and semantic representation, as detailed below.

To ensure comparability across heterogeneous resources and to enable their use in optimization algorithms, raw hardware indicators are converted into a unified normalized representation [36]. Each resource metric *k* is normalized to the interval [0, 1]:

(3) $$R_{k}=\frac{x_{k}-x_{k}^{\min}}{x_{k}^{\max}-x_{k}^{\min}},$$

where *x_k_* denotes the measured value.

A multi-dimensional hardware resource vector for each edge node is subsequently formed as:

(4) $$R^{*}=\left[R_{cpu},R_{gpu},R_{mem},R_{bw}\right],$$

which is used by the compression decision module in later stages.

To enable the compression algorithm to effectively capture the structural characteristics of heterogeneous hardware environments, a hierarchical encoding scheme is employed. Hardware resources are organized into multiple semantic layers, including a compute layer (CPU/GPU capabilities), a memory layer (RAM capacity and cache hierarchy), and an I/O layer (network bandwidth and storage throughput). Based on this decomposition, a hierarchical hardware representation is constructed as $R^{*}=\left\{R_{comp}^{*},R_{mem}^{*},R_{io}^{*}\right\}$, providing a structured and semantically coherent description of hardware resources that can be readily utilized by the downstream decision engine.

Similarly to the task-level abstraction in the previous subsection, each edge node is represented as a directed graph *G* = (*V*, *E*), where the vertex set *V* corresponds to hardware resource entities (CPU, GPU, RAM, and storage), and the edge set *E* denotes resource interactions or dependency relations. This graph-based representation supports compression-strategy search, runtime optimization, and hierarchical bottleneck identification, providing a structural foundation for analyzing hardware coupling patterns within heterogeneous edge environments.

A device ontology is further employed to formally define the semantic attributes of hardware resources, as shown in Listing 1. This structured semantic representation enables the decision engine to perform constraint reasoning, infer adaptive capabilities under varying workloads, and conduct cross-device comparisons during compression strategy generation.


**Listing 1.** Edge device ontology.Edge Device ├── ComputeUnit │  ├── CPU: core, frequency, utilization, SIMD │  └── GPU: SM, freq, VRAM, tensor_support ├── MemoryUnit: size, bandwidth ├── NetworkUnit: bandwidth, latency, loss └── StorageUnit: rnd_read, rnd_write


It should be noted that the device ontology proposed in this paper is intentionally designed as a lightweight and compression-oriented abstraction, rather than a full reuse of existing hardware ontologies. Although several general-purpose ontologies have been proposed for system modeling and semantic interoperability, they typically include a wide range of attributes that are not directly relevant to adaptive model compression and may introduce unnecessary complexity for real-time decision-making on edge devices. In contrast, the proposed ontology focuses on a minimal set of compression-relevant hardware attributes and interaction relationships, enabling efficient reasoning, fast embedding construction, and seamless integration with learning-based optimization modules. The ontology is designed to be extensible and can be aligned with existing standards when needed.

### 4.3. Multi-Strategy Compression Candidate Pool

This subsection describes how a composable, extensible, and searchable compression strategy candidate pool (CSCP) is constructed by integrating diverse compression principles, algorithmic classes, and structural properties of neural models. The CSCP serves as a foundational component for the adaptive compression decision engine, since the quality and completeness of the candidate strategies directly determine the effectiveness of model adaptation on heterogeneous edge devices and the attainable performance after deployment.

To accommodate diverse model architectures and heterogeneous hardware platforms, the candidate pool is organized hierarchically according to compression paradigms, encompassing four major categories of lightweight techniques:

(1) Pruning-based strategies. This layer covers various pruning approaches from structural to parameter levels, including structured pruning, unstructured weight sparsification, importance-score-based pruning, and dynamic pruning strategies that adjust pruning rates at runtime. Each method is encoded into the candidate pool using a triplet (method, granularity, ratio).
(2) Quantization-based strategies. Quantization schemes are selected based on device computational characteristics and may include symmetric or asymmetric quantization, weight/activation/layer-wise quantization, quantization-aware training or post-training quantization, and per-layer mixed-precision search. Strategies are represented as tuples (bitwidth, calibration_method, per_layer_flag).
(3) Low-rank decomposition strategies. Suitable for matrix-intensive models such as Transformers, this layer includes SVD decomposition for fully connected layers, CP/Tucker decomposition for convolutional kernels, and block-wise low-rank decomposition. Each strategy is parameterized as (rank, block_size).
(4) Knowledge distillation strategies. Designed to preserve accuracy under high compression ratios, this layer supports logits distillation, feature distillation, attention-map distillation, and response-based versus hint-based distillation approaches. Strategies are encoded using a tuple (loss_type, temperature, loss_weight).

This hierarchical and parameterized encoding enables systematic, composable, and extensible representation of compression strategies within the adaptive decision engine.

To enable combinatorial search of compression strategies, each technique is abstracted as an independent strategy unit (*SU*) with a standardized interface:

(5) $$SU=\left\{type,params,\cos t,effect,compatibility\right\},$$

where *type* denotes the category of the method (pruning, quantization, decomposition, or distillation), *params* encodes method-specific hyperparameters (e.g., pruning ratio, bitwidth, rank), *cost* represents estimated resource consumption or improvement, *effect* captures the predicted impact on accuracy via empirical models, and *compatibility* indicates feasibility of combining this unit with other strategy units. This abstraction enables the compression strategies to be formally represented, mathematically manipulated, and efficiently searched within the adaptive decision method.

To enable the decision engine to search for optimal strategy combinations within a large-scale compression space, parameter domain sampling and subspace construction are performed. Critical parameters are discretized for tractable exploration. The overall strategy space is defined as the Cartesian product:

(6) S=P×Q×D×K,

where *P* is the set of pruning strategies, *Q* is the set of quantization strategies, *D* represents low-rank decomposition strategies, and *K* denotes knowledge distillation strategies. This structured space provides a well-defined domain for exploration by RL or BO algorithms.

It is worth noting that the compression strategy candidate pool also serves as a form of state-space reduction, which is a well-studied problem in cyber-physical systems. Conceptually, this idea is related to the notion of basis marking in labeled Petri nets, where a reduced set of representative states is constructed to preserve system predictability and key behavioral properties while avoiding exhaustive state enumeration [37]. While the proposed framework does not explicitly model system dynamics using Petri nets, the candidate pool plays a similar role by constraining the decision space of compression strategies, thereby improving decision efficiency and stability in complex runtime environments.

### 4.4. Hybrid RL and BO Decision Engine

**Figure 3:**
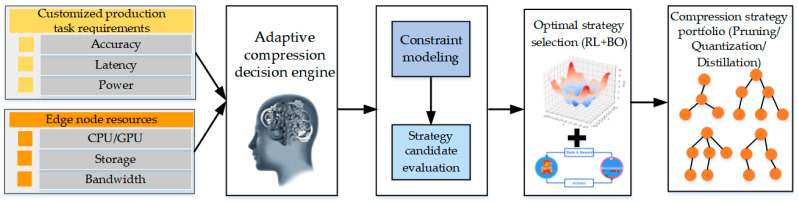
Schematic diagram of the adaptive compression decision engine.

The adaptive compression decision engine is designed to select or compose optimal model compression strategies, including pruning, quantization, and knowledge distillation, according to task-level constraints such as latency, accuracy, and energy consumption and hardware resources such as memory capacity and computational capability. RL and BO are integrated to enable dynamic and constraint-aware compression decisions within the system. The schematic diagram of the adaptive compression decision engine is shown in Figure 3.

Assume that the system contains *N* compression strategies, each associated with a distinct set of hyperparameters represented by a vector $s_{i}\in S_{i}$ for the *i*-th strategy. The overall decision space is defined as:

(7) $$s=\left[s_{1},s_{2},\cdots ,s_{N}\right]\in S_{1}\times S_{2}\times \cdots \times S_{N}.$$

The combined compression strategy is expressed as:

(8) $$s=\arg\underset{s\in S}{\max}R\left(s,T_{s},H\right),$$

where $R\left(s,T_{s},H\right)$ denotes the performance score of strategy s under the task-specific constraints $T_{s}$ and the resource conditions H of the edge node.

The performance of the proposed method is evaluated through a scoring function that jointly considers model accuracy A, inference latency Ι, and memory usage Μ. The aggregated performance model is formulated as:

(9) $$R\left(s,T_{s},H\right)=\alpha A\left(s\right)-\lambda Ι\left(s,H\right)-\mu Μ\left(s,H\right),$$

where α, λ and μ are weighting coefficients that can be adjusted according to the requirements of customized manufacturing tasks.

Within the RL framework, the selection of compression strategies is modeled as a Markov decision process. The optimal strategy distribution is defined as:

(10) $$\pi^{*}\left(\left.s\right|T_{s},H\right)=\arg\underset{\pi }{\max}\mathbb{Z}\left[\sum_{t=0}^{T}\left.\varphi^{t}r_{t}\right|\pi \right],$$

where $r_{t}=R\left(s_{t},T_{s},H\right)$ denotes the reward at step, φ is the discount factor, and $\pi^{*}$ represents the optimal adaptive compression strategy obtained by the proposed method.

In addition to RL, BO is integrated to guide efficient exploration. A Gaussian process is used to model the performance function $R\left(s\right)$, and the next candidate strategy is selected through an acquisition function. The Bayesian update is expressed as:

(11) $$s_{t+1}=\arg\underset{s\in S}{\max}ΕΙ\left(s\right),$$

(12) $$ΕΙ\left(s\right)=\mathbb{Z}\left[\max\left(0,R\left(s\right)-R_{s}^{*}\right)\right],$$

where $R_{s}^{*}$ denotes the best observed performance and $ΕΙ\left(s\right)$ is the expected improvement, balancing exploration and exploitation.

The proposed method performs rapid search using BO during the offline phase to identify a set of candidate compression strategies, while RL is employed during deployment to dynamically schedule strategies within this candidate set. Algorithm 1 details the adaptive compression strategy search under a hybrid offline and online paradigm, where the offline phase handles candidate strategy generation and policy initialization, and the online phase performs lightweight strategy scheduling and performance monitoring on edge devices.
**Algorithm 1:** Adaptive compression strategy searchInput: Task constraint vector *T*, hardware resource vector R*, candidate strategy pool *S* = {*s*_1_, *s*_2_, …, *s_n_*}, initial model *M*_0_ Output: Optimal compression strategy combination π* 1: Initialize strategy network *π_θ_* and value network *V_φ_* 2: Construct state representation *x*_0_ = Embed(*T*, R*) 3: *M_T_* ← *M*_0_ 4: for *episode* = 1 to *MaxEpisode* do 5:   for *step* = 1 to *MaxStep* do 6:     Select compression action *a_t* ~ *π_θ_*(*a* | *x_t*) 7:     Apply action *a_t* to model → *M_t* = Compress(*M_T_*, *a_t*) 8:     Evaluate *M_t* to obtain: 9:       accuracy *Acc_t*, latency *Lat_t*, energy *E_t* 10:    Compute reward: 11:      *r_t* = *w*_1_·*Acc_t* − *w*_2_·*Lat_t* − *w*_3_·*E_t* 12:    Update state representation *x*_{*t* + 1} = *f*(*M_t*, *T*, R*) 13:    Store transition (*x_t*, *a_t*, *r_t*, *x*_{*t* + 1}) 14:    if termination condition satisfied then 15:      break 16:    end if 17:  end for 18:  Update *π_θ_* and *V_φ_* using collected transitions 19: end for 20: Extract best-performing strategy sequence π* from policy *π_θ_* 21: return π*

The specific steps are as follows. First, input the task constraint vector *T*, the hardware resource vector R*, and the candidate compression strategy set *S*, followed by initializing the policy network, value network, and other relevant state parameters. Second, construct the initial state representation according to *T* and R*, and select the first compression action based on the policy network to generate the preliminary compressed model. Third, invoke the model evaluation function to return the accuracy, latency, and energy metrics, and compute the corresponding reward to guide the policy update. Meanwhile, update the state representation and determine whether the termination conditions are satisfied. Fourth, repeat step 3 to continuously search for better compression strategies until the reward converges or the maximum number of iterations is reached. Then, extract the highest-performing compression strategy sequence as the optimal solution. Finally, the edge node deploys the compressed model and executes real-time inference under the given resource constraints while uploading performance feedback for subsequent iterations. The proposed method is applicable to lightweight model generation in various edge-side target recognition tasks.

The adaptive compression strategy search comprises two phases. In the offline phase, BO is employed to efficiently explore the compression strategy space and construct a compact candidate strategy pool, while the RL policy and value networks are trained or initialized using representative task and hardware profiles on resource rich platforms such as cloud servers or industrial workstations. In the deployment phase, edge nodes do not perform model retraining or weight updates but dynamically select and switch among pre generated compression strategies based on real-time feedback on latency, accuracy, and energy consumption, with performance logs asynchronously uploaded to support potential offline policy refinement.

The proposed system is designed with a modular architecture to facilitate adaptability and extensibility. Compression methods are abstracted as independent strategy modules, each characterized by a set of controllable parameters, expected performance effects, and constraint interfaces. As a result, newly developed compression techniques can be integrated into the framework by registering their parameter spaces and performance feedback channels, without modifying the core decision logic. The decision engine operates on unified task–hardware embeddings and runtime performance indicators, making it largely agnostic to the internal implementation details of individual compression methods. This design enables the framework to evolve alongside advances in model compression research and adapt to future edge deployment requirements.

### 4.5. Closed-Loop Runtime Optimization

**Figure 4:**
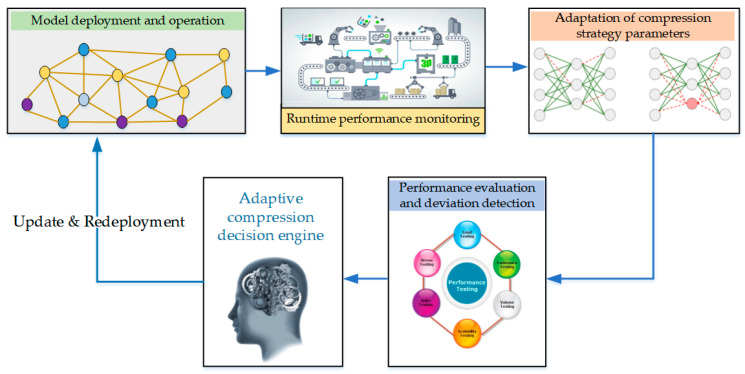
Closed-loop control for iterative optimization.

In practical deployment, the compression strategy must be adjusted dynamically rather than remaining static. Real-time operational indicators, such as inference latency and accuracy degradation, are incorporated into a closed-loop optimization mechanism that continuously updates the selected configuration. This closed-loop process enables the system to maximize performance under hardware resource constraints while preserving task-level accuracy. The structure of the closed-loop control for iterative optimization is shown in Figure 4.

During deployment, the system monitors several key indicators, including inference time $J_{t}$, accuracy degradation $\Delta A_{t}=A_{orig}-A_{comp}$, memory usage $M_{t}$, and energy consumption $E_{t}$. These metrics are represented as a real-time performance vector:

(13) $$y_{t}=\left[J_{t},\Delta A_{t},M_{t},E_{t}\right].$$

The corresponding real-time performance model is expressed as:

(14) $$y_{t}=f\left(s_{t},R^{*}\right),$$

where $s_{t}$ denotes the current compression strategy, R* represents the hardware resource constraints, and denotes the mapping function *f* that relates the strategy–resource pair to the resulting performance.

The objective of the closed-loop optimization is to minimize a weighted combination of inference latency and accuracy degradation. The corresponding loss function is formulated as:

(15) $${ℑ}_{t}=\delta_{1}J_{t}+\delta_{2}\Delta A_{t}+\delta_{3}M_{t}+\delta_{4}E_{t},$$

where the weighting coefficients $\delta_{t}$ reflect the relative importance of each metric and can be dynamically adjusted according to the requirements of specific customized manufacturing scenarios.

Let the current compression ratio be denoted as $\rho_{t}$. The closed-loop optimization updates this ratio through gradient descent or strategy search, yielding the following update rule:

(16) $$\rho_{t+1}=\rho_{t}-\eta \frac{\partial {ℑ}_{t}}{\partial \rho_{t}},$$

where η represents the learning rate, and $\frac{\partial {ℑ}_{t}}{\partial \rho_{t}}$ captures the sensitivity of latency, accuracy, and resource usage with respect to changes in the compression ratio.

The closed-loop optimization adjusts the compression strategy using feedback from real-time performance metrics. The strategy update model is expressed as:

(17) $$s_{t+1}=s_{t}+\Delta s_{t},$$

(18) $$\Delta s_{t}=g\left(y_{t},T_{s},R^{*}\right),$$

where *g* represents the mapping function that generates the adjustment magnitude based on monitored metrics, task-specific constraints, and edge node resources.

The proposed closed-loop runtime optimization mechanism monitors multiple indicators, including inference latency, detection accuracy, and power consumption. When multiple constraints are violated simultaneously, a priority-aware decision strategy is adopted. In particular, latency is treated as a hard constraint, as real-time responsiveness is critical for industrial production lines. Accuracy and power consumption are considered soft constraints and are optimized after latency requirements are satisfied. This hierarchical decision logic ensures system stability and prevents unacceptable real-time performance degradation.

The adjustment step size of compression parameters is dynamically adapted rather than fixed. The step size is determined based on the normalized deviation between the current runtime indicators and their target thresholds. Larger step sizes are applied when violations are significant to accelerate convergence, while smaller steps are used near the feasible operating region to avoid oscillation and excessive accuracy loss. In addition, historical feedback from previous optimization iterations is incorporated to further stabilize the adjustment process under fluctuating workloads and device-level performance variations.

By iteratively updating $s_{t}$ and $\rho_{t}$, the edge computing system in a customized manufacturing environment can perform adaptive compression during inference, achieving a balanced trade-off among latency, accuracy, and resource utilization. When a new compression strategy is selected during runtime, the system does not perform backpropagation or on-device retraining to recover accuracy. Instead, all candidate compression strategies are generated and optionally fine-tuned offline, ensuring that each strategy corresponds to a pre-validated model configuration. Runtime adaptation is therefore achieved through lightweight strategy switching rather than computationally expensive optimization, making the approach suitable for resource-constrained edge devices. The details of the closed-loop deployment-time compression adjustment are given in Algorithm 2.
**Algorithm 2:** Closed-loop deployment-time compression adjustmentInput: Deployed model *M_T_*, constraint thresholds (*Acc*_*min*, *Lat*_*max*), adjustment operator Adjust() Output: Adaptively optimized deployed model M* 1: Initialize performance buffer *B* ← ∅ 2: while system is running do 3:   Monitor real-time performance: 4:     *Acc_cur* = MeasureAccuracy(*M_T_*) 5:     *Lat_cur* = MeasureLatency(*M_T_*) 6:   Append (*Acc_cur*, *Lat_cur*) to buffer *B* 7:   if Mean(*B.acc*) < *Acc_min* then 8:     // Accuracy is degraded 9:     *M_T_* ← Adjust(*M_T_*, direction = “decompress”) 10:  else if Mean(*B.lat*) > *Lat_max* then 11:    // Latency violation 12:     *M_T_* ← Adjust(*M_T_*, direction = “compress”) 13:  end if 14:  if Convergence(*B*) == true then 15:     break 16:  end if 17: end while 18: return M*

First, the deployed model *M_T_* continuously ran on the system while the real-time performance was monitored. The current accuracy *Acc_cur* and latency *Lat_cur* were measured and appended to the performance buffer *B*. Second, the mean accuracy Mean(*B.acc*) was compared with the minimum accuracy threshold *Acc_min*, and if the accuracy was below the threshold, the model was adjusted in the decompression direction using the adjustment operator Adjust(). Otherwise, the mean latency Mean(*B.lat*) was compared with the maximum latency threshold *Lat_max*, and if the latency exceeded the limit, the model was adjusted in the compression direction. Third, the convergence condition of the buffer *B* was evaluated; if the performance metrics in *B* satisfied convergence, the loop terminated. Finally, the adaptively optimized model M* was returned, completing the real-time closed-loop adjustment of compression and decompression to balance accuracy and latency.

## 5. Experimental Results

This section presents a comprehensive evaluation of the proposed adaptive multi-strategy compression method across multiple models, hardware platforms, and real-world industrial scenarios. The experiments are designed to demonstrate the method’s effectiveness in terms of accuracy preservation, latency reduction, memory efficiency, energy consumption, and adaptiveness under dynamic manufacturing tasks. The evaluation includes: (1) Four heterogeneous edge-computing platforms; (2) Four baseline compression methods; (3) Ablation studies for each component (RL, BO, closed-loop feedback); (4) Cross-device transferability and robustness tests.

### 5.1. Experimental Setup

The experimental setup utilized an industrial dataset sourced from a high-speed packaging line, comprising 1200 images with four annotated anomaly types (chip, stain, misalignment, deformation). To further verify the generalization capability of the proposed adaptive compression method, we conducted additional experiments on the COCO 2017 dataset, which contains diverse object categories, complex backgrounds, and significant scale variations. Compared with the industrial dataset, COCO poses a more challenging and heterogeneous detection environment, making it suitable for evaluating robustness across data distributions. To meet the real-time operational demands, performance thresholds were mandated at ≥95% accuracy and ≤50 ms inference latency. The edge computing platform used in this study includes several NVIDIA Jetson modules (NVIDIA Corporation, Santa Clara, CA, USA) representing a range of typical deployment environments in manufacturing. The details of the edge devices are given in Table 1. Collectively, these devices cover a spectrum from low-power to mid-high-performance edge computing scenarios commonly encountered in industrial applications.

The evaluation includes four representative categories of mainstream model compression methods: static pruning (SP), which applies a fixed pruning ratio; static quantization (SQ), referring to 8-bit post-training quantization; knowledge distillation (KD); and RL-based pruning (RL-P), which adopts reinforcement learning without Bayesian optimization or any closed-loop mechanism. The proposed method is denoted as AMS-RLBO. Two widely used industrial models are selected for evaluation: YOLOv5s for object detection, which is suitable for edge deployment and commonly applied in production-line visual inspection, and ResNet-50 for image classification. To further validate the generality of the proposed adaptive compression framework and address recent advances in object detection models, we additionally conducted comparative experiments using YOLOv8n, a representative lightweight model from newer YOLO generations, which is widely considered more accurate and structurally advanced than YOLOv5s. The YOLOv5s and YOLOv8n baseline models are trained in PyTorch 1.13.1 for 300 epochs. The BO module is executed for 50 iterations, while the RL agent is updated every 50 inference samples. The learning rate for the RL component is set to 0.0003, and the teacher temperature is configured as τ = 2.0. Energy consumption is measured using the NVIDIA tegrastats tool. All experiments were repeated five times under identical settings. The reported results are presented as mean ± standard deviation. To evaluate the statistical significance of performance differences between AMS-RLBO and baseline methods, paired two-sided t-tests were conducted. Improvements with *p* < 0.05 are considered statistically significant.

### 5.2. Results and Discussion

(1) Compression performance for YOLOv5s: The compression performance of YOLOv5s was evaluated against the four baseline methods described in the previous subsection. The results are shown in Table 2. The main findings demonstrate that AMS-RLBO achieves a 58.6% reduction in latency while maintaining accuracy ≥ 95%. The dynamic strategy ensures stable inference under varying illumination conditions and target size changes, with a 30% decrease in power consumption, demonstrating strong suitability for long-term operations. Traditional compression methods lack the capability to sense and respond to industrial settings variations, leading to degraded accuracy or increased latency. In contrast, the closed-loop mechanism incorporated in AMS-RLBO ensures that the system compensates for performance deviations by promptly refining the compression ratio or modifying quantization and distillation parameters. This guarantees sustained operational reliability, which is essential for inline inspection and real-time decision-making on production lines.

The proposed AMS-RLBO method introduces a slight accuracy reduction (approximately 0.5% mAP) compared with the uncompressed source model. This degradation mainly arises from structured pruning and low-bit quantization, which reduce parameter redundancy and numerical precision. Although knowledge distillation effectively recovers most of the lost accuracy, a small residual gap remains due to the constrained model capacity under aggressive compression. In industrial inspection scenarios, such a minor accuracy loss is generally acceptable given the substantial improvements in inference latency, memory footprint, and power consumption. In our experiments, AMS-RLBO reduces latency by more than 50% while maintaining accuracy well above practical industrial requirements, representing a favorable trade-off for real-time edge deployment.

**Figure 5:**
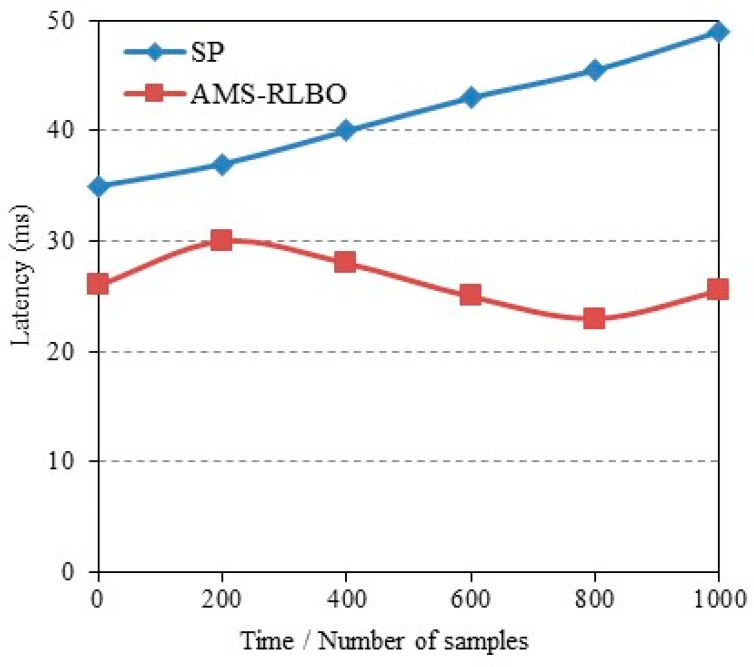
Comparison of inference latency between AMS-RLBO and SP.

(2) Compression performance comparison between YOLOv5s and YOLOv8n: The experiments were performed on an industrial dataset using the Jetson Nano platform to ensure a fair comparison under strict edge resource constraints. Both YOLOv5s and YOLOv8n were evaluated under identical training settings, compression strategies, and hardware conditions. For YOLOv8, the official nano-scale architecture (YOLOv8n) was selected to reflect realistic edge deployment scenarios. The results are shown in Table 3. While YOLOv8n achieves higher baseline accuracy than YOLOv5s without compression, it exhibits increased sensitivity to aggressive compression strategies, particularly structured pruning and low-bit quantization. As a result, its latency reduction under static compression is limited, and accuracy degradation becomes more pronounced. In contrast, the proposed adaptive multi-strategy compression method effectively balances accuracy and efficiency for both architectures, demonstrating consistent latency reduction and improved compression robustness. These results indicate that although newer YOLO architectures provide stronger representational capacity, their increased structural complexity makes them less tolerant to static compression on low-power edge devices. The proposed adaptive method mitigates this issue by dynamically adjusting compression strategies based on runtime feedback, thereby maintaining stable performance across different model generations.

(3) Evaluation on public dataset (COCO): In this experiment, YOLOv5s was trained following the standard COCO training protocol, and the proposed adaptive compression method was applied under the same hardware constraints as the industrial experiments. The evaluation was conducted on the COCO 2017 validation set using the mAP@[0.5:0.95] metric. Baseline methods, including static pruning and static quantization, were also evaluated for comparison. All methods were evaluated under identical hardware and inference settings. The results are shown in Table 4. Although the baseline accuracy on COCO is lower than that of the industrial dataset due to increased task complexity, the proposed method consistently achieves significant latency reduction while preserving detection accuracy. This demonstrates that the adaptive compression strategy generalizes well beyond domain-specific industrial data and remains effective under complex real-world visual scenarios.

(4) Compression performance for ResNet-50: Experiments on industrial dataset exhibit consistent trends across classification settings. As shown in Table 5, AMS-RLBO achieves the strongest overall performance when compressing ResNet-50 on industrial dataset. Compared with SP, SQ, and the RL-P, AMS-RLBO preserves higher Top-1 accuracy while delivering the highest speedup and the largest reduction in parameters. These results indicate that AMS-RLBO maintains accuracy more effectively under aggressive compression and provides consistently stronger overall performance on classification tasks. One of the most striking observations from the experiments is the method’s ability to maintain stable performance under real-world customized manufacturing conditions. Unlike conventional compression strategies that are designed offline and remain static during deployment, the proposed method operates as a self-regulating system. The integration of RL-based online control and BO-assisted global optimization enables continuous strategy adjustment in response to fluctuating workloads, changing scene conditions, and device-level performance drift.

(5) Edge-hardware heterogeneity and generalization: Customized manufacturing environments often involve a mixture of edge hardware platforms due to incremental upgrades and different production line ages. The proposed method demonstrates strong cross-device generalization, as evidenced by consistent performance on Jetson Nano, TX2, NX, and Orin Nano. The results are shown in Table 6. AMS-RLBO consistently maintained optimal performance across all tested hardware platforms, which underscores the critical importance of a hardware-aware strategy. Two key factors support the generalization capability of AMS-RLBO. First, hardware-aware strategy modeling provides the RL agent with explicit information about device constraints, enabling it to make decisions that remain compatible with the available computational resources. Second, surrogate-based hardware performance prediction uses lightweight regression models trained on offline profiling data to estimate latency, memory usage, and energy cost for candidate compression strategies on new devices. This supplies hardware-specific guidance without requiring retraining. Together, these components allow AMS-RLBO to scale down efficiently on resource-limited edge devices and scale up to fully utilize high-performance platforms, while avoiding manual intervention.

(6) Ablation study: To quantify the contribution of each component in the method, three ablation variants are evaluated on industrial dataset: removing BO (No BO), removing the RL module (No RL), and disabling the closed-loop feedback mechanism (No Feedback). The results in Table 7 show that excluding BO leads to reduced mAP and higher latency, indicating its role in providing global strategic guidance. Removing RL further increases latency and yields the lowest mAP among the variants, reflecting the importance of real-time adaptation. Disabling feedback improves neither accuracy nor latency compared with the full system, highlighting its role in maintaining long-term stability. The complete AMS-RLBO configuration achieves the highest mAP and the lowest latency, demonstrating that all three components jointly contribute to performance gains.

(7) Runtime adaptiveness test: A runtime evaluation was conducted to simulate conditions commonly encountered on industrial production lines, including illumination changes, partial occlusions, and batch switching across different product types. Latency was recorded after every fixed number of processed samples to assess stability over time. The results are shown in Figure 5. The comparison between AMS-RLBO and Static Pruning shows a clear contrast in adaptiveness. The Static Pruning baseline exhibited a gradual increase in latency as the environment changed, rising from an initially low level to noticeably higher values. In contrast, AMS-RLBO maintained a stable latency profile throughout the entire evaluation period, consistently operating within a narrow range despite variations in scene conditions and workload. This result indicates that the adaptive mechanism effectively mitigated performance drift and ensured reliable real-time behavior.

(8) Energy efficiency: An evaluation on the Jetson Nano platform was conducted to assess the energy benefits of adaptive compression. The results are shown in Table 8. The results show that AMS-RLBO achieved the lowest average power consumption and the smallest cumulative energy usage among all compared methods. SP and the RL-based pruning baseline reduced energy consumption to some extent, but both remained less effective than the adaptive strategy. The original uncompressed model exhibited the highest power draw. Overall, the dynamic compression behavior of AMS-RLBO enabled more efficient use of limited energy resources on edge devices, demonstrating clear advantages for long-duration industrial deployments.

The experimental findings demonstrate several strengths of AMS-RLBO across the evaluated datasets and hardware platforms. The method consistently maintains approximately 95% accuracy, indicating strong robustness under compression. Compared with static compression strategies, latency is reduced by 30% to 60%, reflecting substantial efficiency gains. It is worth noting that AMS-RLBO is not fundamentally constrained to operate below the accuracy of the source model. With less aggressive compression ratios, stronger teacher supervision, or task-specific fine-tuning, the compressed model can approach or even match the original accuracy. Moreover, the adaptive compression process may act as an implicit regularization mechanism, which has the potential to improve generalization under certain conditions. Exploring accuracy recovery strategies and accuracy–efficiency Pareto optimization will be an important direction for future work.

Since runtime adaptation in the proposed framework does not involve backpropagation or parameter optimization, its impact on system latency is minimal. Strategy switching primarily consists of loading pre-compressed model parameters or activating alternative execution configurations, which introduces negligible overhead compared with inference latency. As a result, the framework maintains stable real-time performance during adaptation phases, which is critical for industrial production environments. These observations suggest that more complex architectures, while powerful, are generally less tolerant to aggressive compression on low-power edge devices, further highlighting the necessity of adaptive and feedback-driven optimization mechanisms. The consistent performance gains observed on both the industrial dataset and the public COCO benchmark indicate that the proposed adaptive compression framework does not rely on domain-specific characteristics and thus demonstrates strong generalization capability across different data distributions and task complexities. The method also exhibits strong cross-device generalizability, adapting to diverse edge platforms without requiring retraining. In industrial environments characterized by continuous operation, varying workpieces, and noise disturbances, the system remains stable and dynamically adjusts its strategies. The closed-loop mechanism further enhances long-term reliability by preventing accuracy degradation over time.

In many industrial applications, retraining deployed models is constrained by data availability, system downtime, and operational safety requirements. The proposed method explicitly accounts for these limitations by focusing on post hoc compression and runtime configuration adaptation. As a result, AMS-RLBO can be deployed on existing models and continuously optimized without interrupting production processes. When retraining is feasible, the framework can optionally incorporate lightweight fine-tuning to further improve accuracy, but such retraining is not a prerequisite for effective operation. While recent AutoML-based compression frameworks such as Tiny Anomaly Compressor (TAC) have demonstrated strong potential for edge deployment, they often require extensive search time, large-scale training data, or cloud-level computational resources. In contrast, this work focuses on adaptive compression under strict industrial constraints, where rapid deployment, predictable overhead, and hardware awareness are prioritized. Therefore, the experimental comparisons emphasize widely used and computationally efficient baselines. Extending the proposed framework to incorporate AutoML-based compression strategies will be explored in future work.

## 6. Conclusions

In this paper, an adaptive multi-strategy model compression method was proposed for lightweight AI deployment in heterogeneous edge environments within customized manufacturing. Task-requirement analysis, hardware-aware resource modeling, and a unified set of pruning, quantization, low-rank decomposition, and knowledge distillation strategies were integrated, allowing the limitations of static compression approaches to be addressed. A hybrid RL and BO decision engine was employed to support broad exploration and fine-grained online adjustment, and a closed-loop feedback mechanism was used to maintain stable accuracy and latency under varying illumination, product changes, and device-performance drift. Through evaluations on an industrial dataset, it was observed that model accuracy was preserved while efficiency and energy usage were improved across multiple edge platforms, demonstrating strong cross-device generalization without the need for retraining. The method was established as a practical and robust foundation for edge intelligence in modern manufacturing, and future extensions were suggested for neural architecture search, federated edge adaptation, and transformer-based compression.

## Figures and Tables

**Table 1:** Four heterogeneous edge-computing platforms.

Edge Device	CUDA Cores	FP32 Performance	Memory (GB)	Power Consumption (W)
Jetson Nano	128	472 GFLOPS	4	10
Jetson TX2	256	1.3 TFLOPS	8	15
Jetson Xavier NX	384	21 TOPS	8	15
Jetson Orin Nano	1024	40 TOPS	16	15

**Table 2:** Performance comparison of compression results for YOLOv5s model trained on the industrial dataset (Jetson Nano, mean ± std, *n* = 5).

Method	mAP (%)	Latency (ms)	Memory Usage (MB)	Power Consumption (W)	*p*-Value (vs. SP)
Source model	95.7 ± 0.2	68.4 ± 1.5	612 ± 8	10.2 ± 0.3	-
SP (40%)	92.1 ± 0.4	45.2 ± 1.3	381 ± 6	8.6 ± 0.2	-
SQ (8 bit)	93.3 ± 0.3	41.9 ± 1.1	355 ± 5	7.9 ± 0.2	-
KD	94.4 ± 0.3	52.7 ± 1.6	410 ± 7	8.8 ± 0.3	-
RL-P	94.8 ± 0.3	39.5 ± 1.2	332 ± 6	7.5 ± 0.2	-
AMS-RLBO	95.2 ± 0.2	28.3 ± 0.9	301 ± 5	7.1 ± 0.2	0.002

**Table 3:** Performance comparison between YOLOv5s and YOLOv8n under edge deployment (Jetson Nano, Industrial Dataset, mean ± std, *n* = 5).

Model	Method	mAP (%)	Latency (ms)	Memory Usage (MB)	Compression Robustness	*p*-Value
YOLOv5s	Original	95.7 ± 0.2	68.4 ± 1.5	612 ± 8	-	-
YOLOv5s	SP	92.1 ± 0.4	45.2 ± 1.3	381 ± 6	Low	-
YOLOv5s	AMS-RLBO	95.2 ± 0.2	28.3 ± 0.9	301 ± 5	High	0.003
YOLOv8n	Original	97.1 ± 0.2	61.2 ± 1.4	654 ± 9	-	-
YOLOv8n	SP	93.4 ± 0.5	47.8 ± 1.5	420 ± 7	Medium	-
YOLOv8n	AMS-RLBO	96.4 ± 0.3	33.7 ± 1.1	338 ± 6	Medium-High	0.009

**Table 4:** Performance comparison on COCO 2017 validation set (Jetson Nano, mean ± std, *n* = 5).

Method	mAP@[0.5:0.95] (%)	Latency (ms)	Memory Usage (MB)	*p*-Value
Original YOLOv5s	36.8 ± 0.3	64.2 ± 1.6	612 ± 8	-
SP	33.4 ± 0.4	44.7 ± 1.2	389 ± 6	-
SQ	34.1 ± 0.3	40.8 ± 1.1	361 ± 5	-
AMS-RLBO	35.6 ± 0.3	29.8 ± 0.9	312 ± 5	0.012

**Table 5:** Compression performance of ResNet-50 on the industrial dataset (Jetson Nano, mean ± std, *n* = 5).

Method	Top-1 (%)	Speedup Ratio	Parameter Reduction (%)	*p*-Value
Source model	78.4 ± 0.3	1.0×	0	-
SP	74.1 ± 0.5	1.9×	40	-
SQ	75.6 ± 0.4	2.2×	0	-
RL-P	76.2 ± 0.4	2.5×	43	-
AMS-RLBO	77.1 ± 0.3	3.3×	52	0.008

**Table 6:** Performance of across hardware platforms (latency, ms).

Method	Jetson Nano	Jetson TX2	Xavier NX	Orin Nano
SQ	41.9	28.7	14.5	9.1
RL-P	39.5	24.9	13.1	8.2
AMS-RLBO	28.3	19.4	11.7	7.4

**Table 7:** Ablation studies validating the effectiveness of the proposed components.

Method Variants	mAP (%)	Latency (ms)
No BO	94.3	34.9
No RL	93.7	41.3
No Feedback	94.8	32.7
AMS-RLBO	95.2	28.3

**Table 8:** Energy consumption varies across different methods on the Jetson Nano platform.

Method	Average Power Consumption (W)	1 h Accumulated Energy (kJ)
Source model	10.2	36.7
SP	8.6	31.0
RL-P	7.5	27.0
AMS-RLBO	7.1	25.6

## Data Availability

The data presented in this study are available on request from the corresponding author.
